# Mediators of screening uptake in a colorectal cancer screening intervention among Hispanics

**DOI:** 10.1186/s12885-021-09092-w

**Published:** 2022-01-05

**Authors:** Navkiran K. Shokar, Jennifer Salinas, Alok Dwivedi

**Affiliations:** 1 Department of Population Health, Dell Medical School at the University of Texas at Austin, DMS Health Discovery Building, #4.702, 1601 Trinity St., BLDG B STOP Z0500, Austin, TX USA; 2 Department of Molecular and Translational Medicine and Family and Community Medicine, Texas Tech University Health Sciences Center El Paso, 5001 El Paso Drive, El Paso, TX 7990 USA; 3 Department of Molecular and Translational Medicine, Texas Tech University Health Sciences Center El Paso, 5001 El Paso Drive, El Paso, TX 79905 USA

**Keywords:** Colorectal Neoplasms, Mass Screening, Hispanic Americans, Psychological Theory, Health Behavior

## Abstract

**Background:**

Colorectal cancer (CRC) is the second leading cause of cancer deaths in the USA. Although a number of CRC screening tests have been established as being effective for CRC prevention and early detection, rates of CRC screening test completion in the US population remain suboptimal, especially among the uninsured, recent immigrants and Hispanics. In this study, we used a structural equation modelling approach to identify factors influencing screening test completion in a successful CRC screening program that was implemented in an uninsured Hispanic population. This information will enhance our understanding of influences on CRC screening among historically underscreened populations.

**Methods:**

We used generalized structural equation models (SEM) utilizing participant reported information collected through a series of surveys. We identified direct and indirect pathways through which cofactors, CRC knowledge and individual Health Belief Model constructs (perceived benefits, barriers, susceptibility, fatalism and self-efficacy) and a latent psychosocial health construct mediated screening in an effective prospective randomized CRC screening intervention that was tailored for uninsured Hispanic Americans.

**Results:**

Seven hundred twenty-three participants were eligible for inclusion; mean age was 56 years, 79.7% were female, and 98.9% were Hispanic. The total intervention effect was comparable in both models, with both having a direct and indirect effect on screening completion (*n* = 715, Model 1: RC = 2.46 [95% CI: 2.20, 2.71, *p* < 0.001]; *n* = 699, Model 2 RC =2.45, [95% CI: 2.18, 2.72, *p* < 0.001]. In Model 1, 32% of the overall effect was mediated by the latent psychosocial health construct (RC = 0.79, *p* < 0.001) that was in turn mainly influenced by self-efficacy, perceived benefits and fatalism. In Model 2, the most important individual mediators were self-efficacy (RC = 0.24, *p* = 0.013), and fatalism (RC = 0.07, *p* = 0.033).

**Conclusion:**

This study contributes to our understanding of mediators of CRC screening and suggests that targeting self-efficacy, perceived benefits and fatalism could maximize the effectiveness of CRC screening interventions particularly in Hispanic populations.

## Background

Colorectal cancer (CRC) remains the second leading cause of cancer deaths among men and women in the USA, with 147,950 new cases and 53,200 deaths expected in 2020 [1]. CRC incidence and mortality have declined significantly over the last two decades, largely attributed to the increase in screening completion that occurred over the same period [2]. CRC screening with a number of different tests has been established as an effective approach to reducing CRC incidence and mortality, is universally endorsed by major professional organizations in the USA and other countries and is considered standard of care [3–5]. . Specific testing strategies vary by country. In the USA, the screening guidelines recommend testing asymptomatic individuals aged 50–75 years of age with either home stool-based screening, endoscopy-based screening or CT colonography [5]. Current US national CRC screening test completion rates among eligible individuals are 66%, with significant screening disparities among Hispanics, Asian Americans, younger individuals, those with lower educational attainment, lower income, and foreign country of birth. The lowest rates of all are reported by the uninsured (30%) [1] and those without a usual source of care (26.3%) [6].

In an effort to increase the completion of recommended CRC screening tests researchers and program developers have studied health behavior theory-based approaches to target potentially modifiable psychosocial mediators of screening among eligible individuals [7–12]. Most studies have used some or all of the Health Belief Model constructs (HBM) [13] to primarily evaluate correlates of past CRC screening. Few successful theory-based interventions have comprehensively assessed either the relative contribution of different constructs, the effect of changes in HBM constructs, or the simultaneous contribution of different constructs on future CRC screening completion [9, 14]. It is particularly important to address these questions among Hispanic Americans because they are less studied, because their CRC screening uptake is low and their cancer burden is expected to rise with demographic shifts. This type of understanding will help to optimize CRC screening and address CRC screening disparities. The primary aim of this study therefore to use a structural equation modelling approach to examine these questions using data from an effective CRC screening intervention designed to promote CRC screening test completion in a predominantly Hispanic community [15, 16].

## Methods

### The ACCION intervention

The ACCION (Against Colorectal Cancer in our Neighborhoods) intervention was a systematically developed health promotion theory-guided intervention designed to promote CRC screening test completion in a predominantly Hispanic community in the USA that was implemented between March 2012 and March 2015. Institutional Review Board approval was obtained prior to the implementation of the study (protocol # 12027) and all participants completed a written informed consent. This analysis is based on participant reported information collected through three longitudinal surveys during the implementation.

Study eligibility included being due for CRC screening (50–75 years of age and not up to date), being uninsured, having a Texas address, no blood in the stool for the prior three months and no history of CRC. The intervention was delivered by community health workers at partnering community and clinical sites. Program personnel provided education, arranged screening tests, diagnostic testing and navigated participants to follow-up care if needed [5] . Participants were offered guideline recommended screening, if average risk they were offered home stool-based testing; twenty-five participants who were above-average risk (i.e., with a family history of CRC or previous adenomas) were offered colonoscopy testing. All screening and follow up testing costs were covered through grant funding. The population for the study consisted of uninsured individuals recruited from community sites or clinics that promoted the program and allowed the community health workers to approach individuals for potential enrollment into the program. The study was composed of culturally tailored education, screening services and navigation that intervened on CRC knowledge, and Health Belief Model constructs of perceived susceptibility, benefits, barriers, fatalism and self-efficacy. The intervention was effective in improving screening (80.5% in the intervention group versus 17.0% in the control group) [15, 17].

### Conceptual framework

For this study, we created a comprehensive conceptual framework to understand potential influences on screening uptake in the ACCION intervention. It was guided by the Health Belief Model (HBM) and incorporated multiple cofactors, and knowledge. The HBM is the most widely examined intrapersonal theoretical model used to explain and predict screening behavior and to guide the development of screening interventions [13, 18]. For this study we included HBM (psychosocial) constructs that have been associated with past and future CRC screening in the literature [19–22] and were targeted by the intervention [17]. We included knowledge because it is recognized as a necessary pre-requisite for performing a behavior and is associated with CRC screening [19, 20]. According to the HBM and extended HBM, knowledge is independent from other considered psychosocial measures for predicting health behaviors. Our model also included cofactors that have been consistently associated with CRC screening [19–21] such as age, gender, years in the US [23], educational attainment, marital status, perceived health status, CRC family history, CRC awareness, having a regular doctor and receipt of a doctor’s recommendation.

### Study population

The original study population included of 784 participants (467 in the intervention group and 317 in the control group) who were surveyed at three time points for the intervention group (baseline, immediate post intervention and at 6 month follow up), and at two time points for the control group (baseline and 6 month follow up). Demographic data was collected at baseline and HBM constructs (psychosocial measures) were included in the bilingual survey at all time points. Of the 784 original study participants, 723 (92.6%) completed both the baseline and six month follow up survey and were eligible for inclusion in this study. The mean age was 56.8 years, 78.4% were female and 98.7% self-reported as Hispanic. The final sample size for the analyses was 715 for Model 1 and 699 for Model 2.

## Measures and data collection

All measures were available in English and Spanish.

### Outcome measure

The outcome of guideline concordant CRC screening [5, 24] was assessed by self-report at the six month survey with a series of validated questions that determined CRC screening uptake with any of the recommended tests (home stool blood testing, colonoscopy or flexible sigmoidoscopy) [25].

### Considered mediators

#### Psychosocial (HBM) constructs

All HBM construct measures were previously validated and had high internal consistency reliability in this population [16, 25]. We assessed: *perceived susceptibility* (perceptions about the likelihood of developing CRC, four item scale, Cronbach’s α = 0.73), *perceived benefits (*beliefs about the advantages of screening, 10 items, Cronbach’s α = 0.89), and *perceived barriers* (beliefs about obstacles to screening, 11 items, Cronbach’s α = 0.88). *Fatalism* (a belief that things that happen in life are determined by fate) was an additional barrier to screening that is important in minority populations [26, 27] and was measured with a validated 15 item scale [28] (Powe, 1995) (Cronbach’s α = 0.85). *Self-efficacy* (confidence in a person’s ability to perform a behavior) was measured with a 12 item adapted scale with Cronbach’s α = 0.91 [29]. As with all listed previous measures, a high score was indicative of higher level of the construct.

All the considered mediators (knowledge, benefits, barriers, fatalism, susceptibility, and self-efficacy) were measured at multiple time-points. In the intervention group they were measured three times (at baseline prior to giving intervention), immediately post intervention, and at 6 months follow up. In the control group they were measured at two time points (baseline and 6 month follow up). In the analysis, for the intervention group we considered the immediate post education psychosocial measures as mediators and for the control group we considered the baseline measures as mediators. These measures truly support mediation in terms of temporality as they were measured six months before the outcome was assessed.

#### Knowledge

We assessed CRC screening knowledge using a validated 10 item knowledge survey (Cronbach’s α = 0.53) that included: one question about CRC curability if diagnosed early, four questions that covered CRC risk factors, one question covering warning signs, and four questions assessing CRC screening and prevention [25, 29]. The response categories were *true or false* and were coded as correct or incorrect and the score was summed.

#### Cofactors

Cofactors assessed at baseline were age (years), gender(race/ethnicity (Hispanic/non-Hispanic), education (diploma/no diploma), income, marital status (living with a partner/no), years living in the US, awareness of CRC screening (yes/no), family history of CRC (yes/no), having a regular doctor (yes/no) and receipt of a doctor’s recommendation (yes/no) for screening.

### Statistical analysis

#### Hypotheses

We utilized a structural equation modelling approach to understand the pathways though which the intervention led to screening completion among Hispanic individuals. Our measurement model was developed using the extended HBM proposed by Orji et al. [30] and exploratory factor analysis. They proposed a measurement model combining psychosocial constructs to amplify their effect on predicting a latent construct which explains health behavior. This was validated on a healthy eating outcome [30] and among different populations [31, 32]. We tested the following hypotheses: 1) the intervention will have a direct effect on screening completion, 2) the intervention will also have an indirect effect on screening completion through the individual psychosocial constructs and the latent psychosocial construct, 3) a higher post- intervention latent psychosocial construct score will be associated with greater screening uptake in the intervention group compared to controls and 4) the latent psychosocial construct score will better predict screening completion than the separate post intervention psychosocial construct scores. We believe this approach is novel, since little is known about how these complex relationships influence CRC screening uptake, particularly in minority groups. This analysis provides a predictive model for CRC screening uptake that could be tested in other high-risk populations. In addition, baseline individual measured psychosocial constructs were considered as confounders in validation analyses.

#### Statistical approach

The separate psychosocial construct scores were obtained by summing the responses for each item for the specific construct (e.g. all barriers items). The latent psychosocial construct score was created by a linear combination of observed individual psychosocial construct scores (barriers, benefits, fatalism, self-efficacy, and susceptibility) that influence the variability. According to the HBM and extended HBM, knowledge is independent from other considered psychosocial measures for predicting health behaviors.

#### Structural equation modeling and analyses

Generalized structural equation models (SEM) were developed to test the four hypotheses. SEM is a multivariate method which allows assessment of the interrelationships of multiple dependent and independent variables by simultaneously developing multiple equations and is typically used to test a proposed conceptual framework. For testing hypotheses 1 and 2, a generalized SEM model (Model 1) was developed to assess the effects of the intervention on the latent psychosocial construct score, knowledge, and screening completion along with the indirect effects of the intervention on screening completion through the latent psychosocial construct and knowledge score. For testing hypothesis 3, a generalized SEM model (Model 2) was developed to assess the effects of the intervention on the separate psychosocial constructs, knowledge, and screening outcome along with indirect effects of the intervention on screening outcome through each psychosocial construct score and knowledge score. Comparing Model 1 with Model 2 tested hypothesis 4, whether the latent psychosocial score model or the individual psychosocial constructs are better predictors of screening outcome. The final SEMs only included the variables which were significant at the 5% level. All non-significant variables were removed to avoid over-parameterization of the model.

MPLUS 7.4 software was used to develop different SEMs. Probit regressions were used to model the screening outcome using a weighted least squares means-adjusted (WLSM) estimation procedure while linear regression models were used to model the quantitative mediators. The regression coefficient for the probit model should be interpreted as probabilities while changes in the observed outcomes in linear regression models.

The path/regression coefficients (RC), related standard errors, and *p*-values obtained from SEMs were used to describe the influence of variables. The total, direct, and indirect effects of the intervention compared to control were estimated through these models and reported along with 95% confidence interval (CI). The model performance was summarized by variability explained (R^2^) for each component of the model. The following criteria were used to assess the goodness of fit of the developed models: (1) a root mean square error of approximation (RMSEA); an RMSEA less than 0.08 is considered an acceptable fit while RMSEA less than 0.05 indicates a good fit. A non-significant *p*-value for RMSEA confirms statistically no difference in estimated RMSEA value from 0.05; (2) a comparative fit index (CFI) and (3) a Tucker-Lewis Index (TLI). The value of 0.90 or higher for CFI and TLI is considered as good fit [33, 34]. The purpose of reporting model fit indices was to verify the parsimony of each model tested. In addition T- rule, residual covariance matrix, and modification indices were also used to assess the quality of model fit and parsimony of the developed models [35]. To validate the direct and indirect effects of the intervention in each model, a separate generalized SEM model was developed by adjusting covariate differences in intervention groups. These models were developed using the maximum likelihood (ML) estimation procedure with a logistic model for binary variables (screening outcome and intervention groups) and a linear regression model for quantitative variables.

#### Sample size

We determined the sample size using the approaches of Muthén & Muthén [36], Wolf et al. [37], and Soper [38] and determined that a sample size of 460 would be more than sufficient to detect a direct path with a coefficient = 0.25 with R^2^ = 0.16 or a coefficient = 0.50 with R^2^ = 0.75, with a corresponding indirect path of 0.06, and 0.25 respectively, with more than 90% power at 5% level of significance without any errors or non-convergence or bias exceeding 5% in the analysis. Further, this sample size would allow the evaluation of a mild to moderate direct effect of 0.5 with one latent variable (the combined psychosocial score) and a maximum of 7 observed variables to detect a significant effect with more than 80% power and at 5% level of significance using a mediation model. Thus, our study sample size of over 700 provided sufficient power to test the hypotheses in this study. As per the rule-of-thumb in SEM, at least 10–15 participants are required to estimate each parameter and accordingly each SEM was developed.

## Results

### Hypothesis 1: the intervention will have a direct effect on screening

Seven hundred twenty-three subjects had an available screening outcome and were included in this data analysis. There were no differences (except for baseline knowledge) in the background and study variables between those who provided 6-month follow-up data and those who did not. 79.7% of the entire sample were female, 98.9% were Hispanic, 89.5% were born in Mexico and the majority (78.6%) had less than a high school education. There were no differences in the estimated effect sizes on the analysis of complete dataset or the imputed dataset due to limited missing cases.

Table 1 shows the total, direct, indirect and specific indirect effects of the intervention, adjusted for baseline differences between groups on screening uptake as compared to control by Model 1 (latent psychosocial construct) and Model 2 (individual psychosocial constructs). There was a significant direct effect of the intervention on screening in both Model 1 (RC = 1.71, 95%CI: 1.25, 2.17, *p* < 0.001) and in Model 2 (RC = 2.15 95%CI: 1.81, 2.50, *p* < 0.001). This supports hypothesis 1 that the intervention had a direct effect on cancer screening uptake.

**Table 1:** Total, direct and indirect effects of the intervention on screening outcome

Screening uptake		
	Coefficient (SE)^**a**^, ***p***-value	Relative measures
**Model 1**		
Total effect	2.457 (0.131),<0.001	
Direct effect	1.709 (0.237),<0.001	0.696
Indirect effect	0.748 (0.197),<0.001	0.304
Via Knowledge	−0.077 (0.03),0.009	−0.031
Via Psychosocial score	0.785 (0.194),<0.001	0.319
Via Knowledge & Psychosocial score	0.041 (0.014),0.003	0.017
**Model 2**		
Total effect	2.445 (0.138),<.001	
Direct effect	2.153 (0.174),<.001	0.881
Indirect effect	0.292 (0.102),0.004	0.119
Via Fatalism	0.072 (0.026),0.046	0.029
Via Knowledge	−0.053 (0.026),0.045	−0.022
Via Self efficacy	0.273 (0.096),0.004	0.112

Model 1: Path analysis with overall psychosocial health construct (benefits, barriers, susceptibility, self-efficacy and fatalism)

Model 2: Path analysis with individual psychosocial construct scores

Some significant variables such as health status, education, doctor recommended were also included in the model 1 while age, gender, health status, education, doctor recommended, heard of colorectal cancer, years in US, and family history of cancer were included in different parts of model 2

*SE* Standard Error

^a^Unstandardized regression coefficient

### Hypothesis 2: the intervention will also have an indirect effect on screening through the individual psychosocial constructs and the latent psychosocial construct score

The intervention also had an indirect effect on screening outcome in both Model 1 (RC = 0.75, 95%CI: 0.36, 1.13, *p* < 0.001) and Model 2 (RC = 0.29, 95%CI: 0.09, 0.49, *p* = 0.004). Thus, our study further supports the hypothesis 2 that there was a significant indirect effect of the intervention through psychosocial mediators.

The total effect of the intervention was comparable in both models: In Model 1, the total effect was 2.46 (95% CI: 2.20, 2.71, *p* < 0.001) compared to control, and in Model 2 it was 2.43, (95% CI: 2.16, 2.71, *p* < 0.001]. However, the indirect effect of intervention was found to be higher in model 1 (30% of the total effect) compared to model 2 (11% of the total effect).

### Hypothesis 3: a higher post intervention latent psychosocial construct score will be associated with greater screening uptake in the intervention group compared to controls

The most significant specific indirect path (RC = 0.79, *p* < 0.001) in structural model 1 was the combined psychosocial score which explained 32% of the total effect. This supports the hypothesis 3 that an increase in latent psychosocial construct score behavior was associated with increased screening uptake in the intervention group compared to control. The most significant specific indirect path in Model 2 was the individual psychosocial constructs of self-efficacy (RC = 0.24, *p* = 0.013, accounting for 10% of the total effect of the intervention) followed by fatalism (RC = 0.07, *p* = 0.033, 3% of the total effect).

### Hypothesis 4: the post intervention latent psychosocial construct score will better predict screening outcome compared to the individual post intervention psychosocial construct scores

The descriptive comparisons of the two models based on standardized effect sizes (direct and indirect) and R^2^ suggests that the latent psychosocial construct score after intervention had a greater predictive ability of screening uptake compared to individual psychosocial constructs after intervention which supports our hypothesis 4.

### Individual psychosocial scores and the latent psychosocial construct

The latent psychosocial construct was positively associated with all individual measured scores except for fatalism (Table 2). Self-efficacy mostly explained the variability in the latent psychosocial construct (58%) followed by benefits (21%). Although the construct was also positively associated with barriers, they only explained 1% of the variability in predicting the psychosocial health construct. Thus, our latent psychosocial score assigns positive weighting to factors (susceptibility, benefits, and self-efficacy) that relate positively with screening uptake while negative weighting to a factor (fatalism) that relates negatively with screening uptake except for barrier which does not contribute much in predicting the latent score.

**Table 2:** Model 1 Path analysis

	**Factor loading (SE)**^**a**^	***p*-value**	**R**^**2**^
**Latent psychosocial health construct**			
Benefit	0.456 (0.0.036)	<0.001	0.208
Barrier	0.143 (0.049)	0.004	0.01
Fatalism	−0.297 (0.040)	<0.001	0.088
Self-Efficacy	0.763 (0.034)	<0.001	0.581
Susceptibility	0.250 (0.041)	<0.001	0.063
	**Coefficient (SE)**^**b**^	***p*-value**	
**Psychosocial health construct score**			0.593
Intervention-education	2.222 (0.202)	<0.001	
Health status-excellent/good/fair	−0.307 (0.118)	0.009	
Doctor recommended CRC-yes	0.335 (0.16)	0.03	
Knowledge	0.267 (0.046)	<0.001	
**Knowledge**			0.05
Intervention-education	0.431 (0.087)	<0.001	
Education-diploma	0.288 (0.119)	0.016	
**Screening uptake**			0.648
Psychosocial score	0.353 (0.076)	<0.001	
Intervention-education	1.709 (0.237)	<0.001	
Knowledge	−0.178 (0.059)	0.003	
**Model fit criteria**	*N* = 715		
	RMSEA = 0.053 (*p* = 0.305)		
	CFI = 0.92		
	TLI = 0.88		

Model 1: Path analysis with psychosocial health construct

*SE* Standard Error, *CRC* Colorectal Cancer, *SE* Standard Error, *R*^2^ Coefficient of Determination, *RMSEA* Root Mean Square Error of Approximation, *CFI* Comparative Fit Index, *TLI* Tucker-Lewis Index

^a^Standardized coefficient

^b^Unstandardized regression coefficient

### Direct intervention effects of the combined psychosocial health score, knowledge, the intervention and baseline variables in model 1

**Fig. 1:**
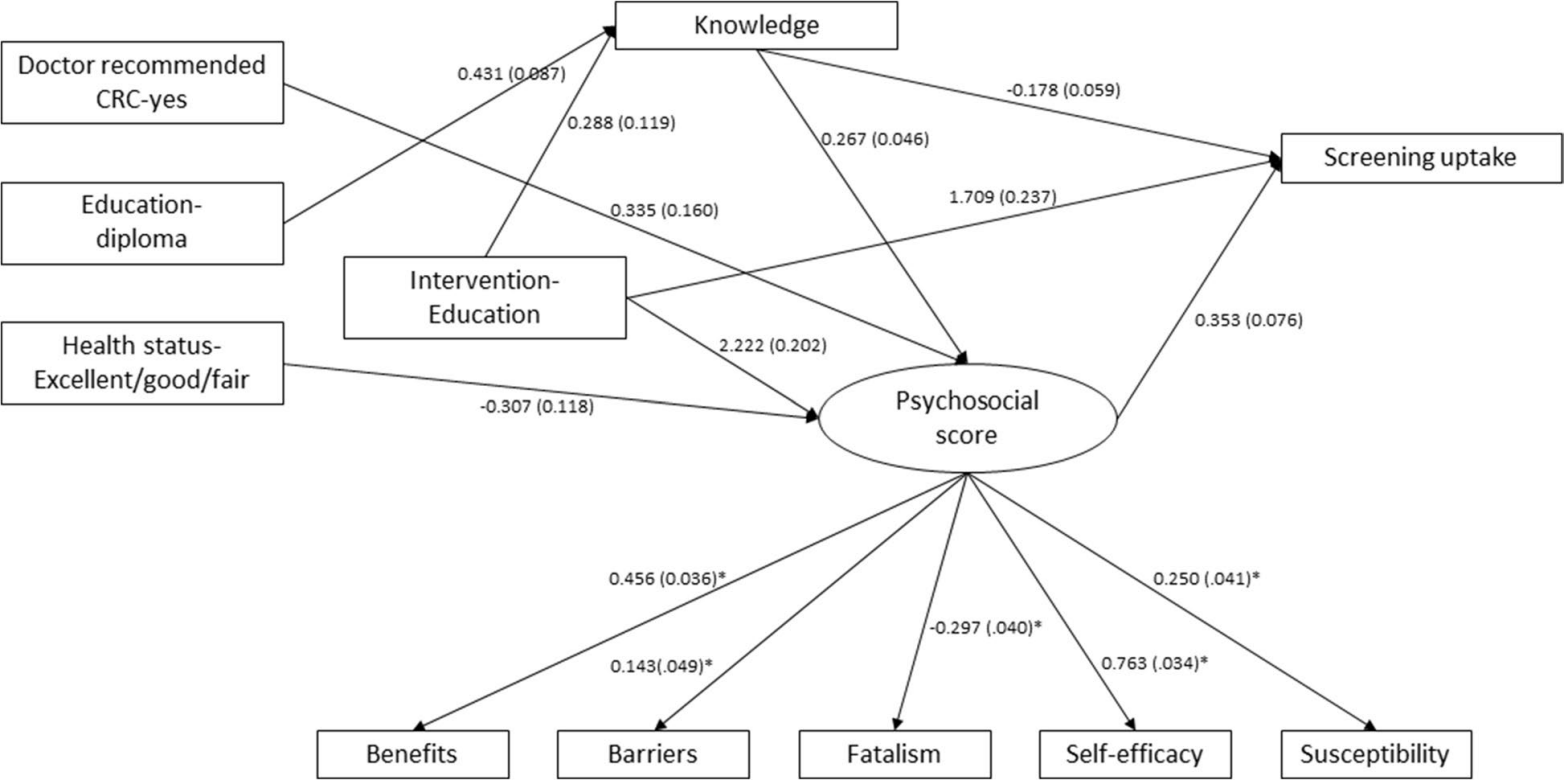
Unstandardized path coefficient (standard error) from the structural equation model analysis using the overall combined psychosocial construct (Model 1). CRC: Colorectal cancer; *standardized coefficient (standard error)

**Fig. 2:**
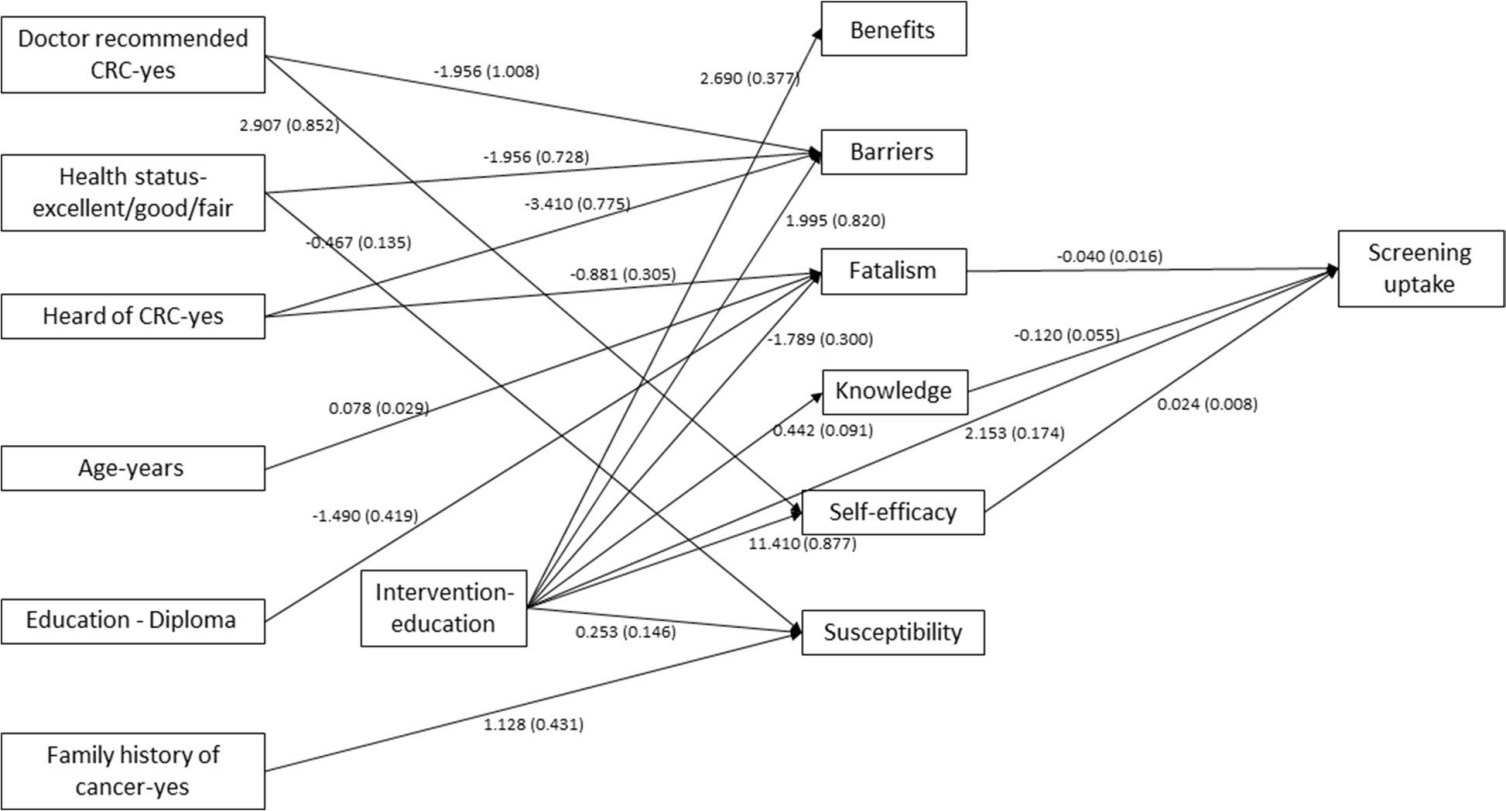
Unstandardized path coefficient (standard error) from the structural equation model analysis model using the individual psychosocial constructs (Model 2). CRC: Colorectal cancer

The path coefficients and their standard errors from the Model 1 analysis are shown in Fig. 1 and Table 2. The path coefficients in Fig. 2 indicate the effect (magnitude) and direction of the associations. The intervention (RC = 1.71, *p* < 0.001), latent psychosocial construct (RC = 0.35, *p* < 0.001), and knowledge (RC = −0.178, *p* = 0.003) all had a direct effect on screening; however, higher knowledge was negatively associated with screening. Overall, 65% of the variability was explained by these three variables in Model 1.

A higher combined psychosocial construct score was associated with receipt of the intervention (RC = 2.22, *p* < 0.001), poor health status (RC = −0.31, *p* = 0.009), increased post education knowledge (RC = 0.27, *p* < 0.001), and a doctor’s recommendation (RC = 0.34, *p* = 0.03). The total effect of the intervention on the combined psychosocial construct was RC = 2.34, *p* < 0.001, mostly through a direct effect. These set of variables explained 59% of the variability in predicting the psychosocial construct in this model.

The intervention significantly influenced the knowledge score (RC = 0.43, *p* < 0.001) as well. The post intervention knowledge score was a positively associated with baseline education level (RC = 0.29, *p* = 0.016). However, only about 5% of the total effect of intervention on improving the psychosocial construct was through improvement of the knowledge score. The RMSEA was observed as 0.053, CFI as 0.92 and TLI as 0.88, reflecting an overall reasonable fit of Model 1. Furthermore, the residual covariance matrix did not indicate any misfit except for residual covariance between self-efficacy and barriers. The inclusion of a covariance term between self-efficacy and barriers in the model 1 did not change any associations (Table 3).

**Table 3:** Residuals for covariances/correlations/residual correlations

	Barrier	Benefit	Fatalism	Knowledge	Self-Efficacy	Susceptibility
Model 1						
Barrier	0					
Benefit	1.802	0				
Fatalism	0.000	−0.399	0			
Knowledge	0.080	0.175	−0.376	0.007		
Self-Efficacy	−7.733	−0.447	1.305	−0.391	0	
Susceptibility	0.601	0.329	0.053	−0.043	0.646	0
Screening	−0.139	−0.028	−0.304	0.003	−0.262	0.100
Model 2						
Barrier	0.017					
Benefit	0.007	0.000				
Fatalism	−0.002	−1.237	−0.048			
Knowledge	0.000	0.000	−0.004	0.000		
Self-Efficacy	−0.083	0.000	−2.148	0.001	−0.007	
Susceptibility	0.000	0.000	−0.181	0.079	0.000	
Screening	0.000	0.000	−0.078	−0.003	−0.011	0.000

Model 1: Path analysis with psychosocial health construct

Model 2: Path analysis with individual psychosocial scores

There were no direct effects of the cofactors on screening uptake in this study. Model 1 was validated by adjusting for differences in cofactors between intervention groups. The results related to validation model 1 were shown in Table 4.

**Table 4:** Model 1 Path analysis after adjusting for confounders

	**Factor loading (SE)**^**a**^	***p*-value**
**Psychosocial health score-construct**		
Benefit	1.117 (0.129)	<0.001
Barrier	0.435 (0.253)	0.085
Fatalism	−0.681 (0.105)	<0.001
Self-Efficacy	4.357 (0.44)	<0.001
Susceptibility	0.219 (0.049)	<0.001
**Psychosocial health score-construct**	**Coefficient (SE)**^**a**^	
Intervention-education	2.44 (0.259)	<0.001
Health status-excellent/good/fair	−0.271 (0.122)	0.026
Doctor recommended CRC-yes	0.420 (0.169)	0.013
Knowledge	0.255 (0.062)	<0.001
**Knowledge**		
Intervention-education	0.426 (0.087)	<0.001
Education-diploma	0.251 (0.103)	0.015
**Screening uptake**		
Psychosocial health score-construct	0.730 (0.237)	0.002
Intervention-education	−0.375 (0.14)	0.007
Knowledge	−0.375 (0.14)	
**Intervention-Education**		
Doctor recommended CRC-yes	−0.515 (0.259)	0.047
Education- diploma	1.161 (0.256)	<0.001
Gender-female	−0.739 (0.25)	0.003
Health status-excellent/good/fair	−0.461 (0.194)	0.017
Heard of CRC	−0.864 (0.211)	<0.001
Baseline barrier	0.064 (0.012)	<0.001
Baseline fatalism	−0.080 (0.027)	0.003
Baseline knowledge	−0.269 (0.079)	0.001
Baseline self-efficacy	0.132 (0.013)	<0.001

Model 1: Path analysis with psychosocial health construct

*SE* Standard Error, *CRC* Colorectal Cancer

^a^Unstandardized regression coefficient

### Direct effects of individual psychosocial constructs, the intervention and baseline variables on screening (model 2)

We further explored the interrelationships of the cofactors, and individual psychosocial constructs and their underlying effects on screening outcome in structural model 2 (see Table 5 and Fig. 2). There was a greater probability of CRC screening in the intervention group compared to control (RC = 2.15, *p* < 0.001). Among the individual psychosocial variables, post intervention self-efficacy (RC = 0.02, *p* = 0.003), and post intervention fatalism (RC = -0.04, *p* < =0.012), had a direct effect on screening uptake. In addition, post intervention knowledge (RC = -0.12, *p* = 0.03) also had a direct effect on screening uptake. This part of the model explained 62% variability in the screening outcome. Comparing performance of this part of Model 1 with Model 2 showed that the combined effect of the post-intervention psychosocial scores had a greater influence on screening uptake (R^2^ = 65%) than the individual psychosocial variables.

**Table 5:** Model 2 Path analysis

	Coefficient (SE)^**a**^	***p***-value	R^**2**^
**Benefit**			0.105
Intervention-education	2.69 (0.377)	<.001	
**Barrier**			0.071
Intervention-education	1.995 (0.820)	0.015	
Doctor recommended CRC-yes	−1.956 (1.008)	0.052	
Health status-excellent/good/fair	−1.956 (0.728)	0.007	
Heard of CRC-yes	−3.410 (0.775)	<.001	
**Fatalism**			0.105
Intervention-education	−1.789 (0.300)	<.001	
Age-years	0.078 (0.029)	0.007	
Education - Diploma	−1.490 (0.419)	<.001	
Heard of CRC-yes	−0.881 (0.305)	0.004	
**Knowledge**			0.035
Intervention-education	0.442 (0.091)	<.001	
**Self-efficacy**			0.351
Intervention-education	11.410 (0.877)	<.001	
Doctor recommended CRC-yes	2.907 (0.852)	0.001	
**Susceptibility**			0.047
Health status-excellent/good/fair	−0.467 (0.135)	0.001	
Family history of cancer-yes	1.128 (0.431)	0.009	
Intervention-education	0.253 (0.146)	0.082	
**Screening uptake**			0.616
Intervention-education	2.153 (0.174)	<.001	
Fatalism	−0.040 (0.016)	0.012	
Knowledge	−0.120 (0.055)	0.030	
Self-efficacy	0.024 (0.008)	0.003	
**Model fit criteria**	*N* = 699		
	RMSEA = 0.014 (*p* = 1)		
	CFI = 0.994		
	TLI = 0.988		

Model 2: Path analysis with individual psychosocial scores

*CRC* Colorectal Cancer, *SE* Standard Error, *OR* Odds Ratio, *R2* Coefficient of Determination, *RMSEA* Root Mean Square Error of Approximation, *CFI* Comparative Fit Index, *TLI* Tucker-Lewis Index

^a^Unstandardized regression coefficient

The intervention had a significant effect on all individual components of the psychosocial construct except for susceptibility. Comparison of standardized effect sizes for different psychosocial scores showed that the maximum effect of the intervention was obtained for self- efficacy (standardized RC = 1.20) followed by benefit (RC = 0.66), fatalism (RC = -0.46), knowledge (RC = 0.38) and barriers (RC = 0.21). There was a significant improvement in perceived self-efficacy in the intervention group compared to controls. In addition, a doctor’s recommendation was also found to be positively associated with self-efficacy (R^2^ = 0.35).

An improved perceived benefit score was obtained in the intervention group and among subjects with higher educational attainment (R^2^ = 11%). Significantly reduced perceived fatalism was observed in the intervention group compared to control (RC = -1.79, *p* < 0.001). Reduced perceived fatalism was also associated with higher education and awareness of CRC while an increase in perceived fatalism score was associated with increasing age (R^2^ = 11%). A higher post-intervention knowledge score was associated with intervention group (R^2^ = 4%). Lower perceived barriers were found to be associated with receipt of a doctor’s recommendation, excellent/good or fair health, and awareness of CRC. However, those who received the intervention reported increased barriers compared to controls (R^2^ = 7%). Perceived susceptibility was higher in the intervention group compared to controls. In addition, higher perceived susceptibility was also associated with a family history of CRC. Surprisingly, individuals with excellent/good or fair health status had a significantly lower perceived susceptibility score compared to their counterparts (variability explained = 5%). A significantly good fit for Model 2 was achieved based on RMSEA <0.05, CFI and TLI >0.9. The residual covariance matrix shown in Table 3 indicates that no misfit occurred in the development of model 2.

### Direct effects of individual psychosocial constructs

Table 6 shows that association among psychosocial variables and the knowledge score. Self-efficacy was found to be correlated with all scores (positively associated with benefits, knowledge and susceptibility while negatively correlated with barriers). Similarly, knowledge was also found to be positively associated with benefits, and self-efficacy while negatively associated with barriers and fatalism. However, knowledge was not associated with susceptibility. Benefits was associated with all scores except for fatalism. Surprisingly benefits and barriers were also positively correlated. In addition to knowledge, self-efficacy, and fatalism were also positively correlated barriers. The significant associations obtained in model 2 remained statistically significant even after adjusting for imbalance in distributions of covariates between intervention groups (Table 7).

**Table 6:** Covariances among psychosocial scores including knowledge score (Model 2)

	Coefficient (SE)^**a**^	***p***-value
**Benefits association with**		
Self-efficacy	5.468 (0.741)	<.001
Susceptibility	0.696 (0.175)	<.001
Barrier	2.587 (1.325)	0.051
**Barrier association with**		
Self-efficacy	−6.383 (3.076)	0.038
**Fatalism association with**		
Barrier	4.776 (1.224)	<.001
**Knowledge association with**		
Self-efficacy	1.167 (0.25)	<.001
Barrier	−1.732 (0.404)	<.001
Fatalism	−0.597 (0.143)	<.001
Benefits	0.579 (0.135)	<.001
**Self-efficacy association with**		
Susceptibility	1.848 (0.411)	<.001

*SE* Standard Error

^a^Unstandardized coefficient

**Table 7:** Model 2 Path analysis after adjusting for confounders

	Coefficient (SE)^**a**^	***p***-value
**Benefit**		
Intervention-education	2.709 (0.299)	<0.001
**Barrier**		
Intervention-education	1.792 (0.739)	0.015
Doctor recommended CRC-yes	−2.031 (1.012)	0.045
Health status-excellent/good/fair	−1.976 (0.711)	0.005
Heard of CRC	−3.559 (0.764)	<0.001
**Fatalism**		
Intervention-education	−1.753 (0.294)	<0.001
Age (years)	0.082 (0.027)	0.003
Education-HS	−1.548 (0.345)	<0.001
Heard of CRC	−0.827 (0.3)	0.006
**Self-Efficacy**		
Intervention-education	11.548 (0.592)	<0.001
Doctor recommended CRC-yes	2.928 (0.819)	<0.001
**Susceptibility**		
Intervention-education	0.285 (0.13)	0.028
Health status-excellent/good/fair	−0.44 (0.128)	0.001
Family history of cancer	1.141 (0.312)	<0.001
**Knowledge**		
Intervention-education	0.445 (0.088)	<0.001
**Screening uptake**		
Intervention-education	3.698 (0.288)	<0.001
Fatalism	−0.067 (0.031)	0.033
Knowledge	−0.259 (0.111)	0.02
Self-efficacy	0.051 (0.019)	0.009
**Intervention-Education**		
Doctor recommended CRC-yes	−0.563 (0.261)	0.031
Education-diploma	1.121 (0.262)	<0.001
Gender-female	−0.698 (0.253)	0.006
Health status-excellent/good/fair	−0.487 (0.197)	0.013
Heard of CRC	−0.85 (0.213)	<0.001
Baseline barrier	0.063 (0.012)	<0.001
Baseline fatalism	−0.08 (0.027)	0.004
Baseline knowledge	−0.258 (0.079)	0.001
Baseline self-efficacy	0.137 (0.014)	<0.001

Model 2: Path analysis with individual psychosocial scores

*SE* Standard Error, *CRC* Colorectal Cancer

^a^Unstandardized regression coefficient

## Discussion

Our study provides an important contribution to what is known about mediators of CRC screening in prospective studies in any population and especially among Hispanics. We utilized a multivariate approach (SEM) to explain the underlying mechanisms through which an effective culturally tailored and theory-based intervention exerted its effect on screening uptake. Our findings demonstrate that the intervention had both a direct and indirect effect on screening completion, with a third of the overall effect being mediated by a latent combined psychosocial score that was in turn mainly influenced by self-efficacy, perceived benefits and fatalism. This suggests that both unobserved mediators and observed psychosocial constructs were crucial for CRC screening uptake in our study population. Furthermore, the combined overall psychosocial score mediated a slightly greater proportion of the effect than when each HBM construct was considered separately perhaps suggesting that interactions among the constructs play a role in improving screening outcome. The combined overall psychosocial score was influenced primarily by self-efficacy, perceived benefits and fatalism, and their interactions along with knowledge were found to be the major modifiable mediators of screening. Although previous studies did not use our approach, the mediators we identified have also been identified as predictors of future screening in prior work.

Our findings suggest that self-efficacy should be a major target for interventions and this is consistent with positive associations with screening reported in other prospective studies [10, 14, 39, 40], and may be especially important for repeat screening, or when organized screening support may be minimal [14]. In addition, our findings also suggest that perceived benefits should be targeted for interventions and this is also consistent with previous literature [19, 20]. We examined fatalism as a specific type of barrier because of its importance among Hispanics and other minorities and demonstrated its significant role in mediating the intervention effect in this Hispanic sample. Previous work has demonstrated that fatalism may not be an intractable cultural belief, since it can be influenced by intervention [16] suggesting it could be an intervention target. We, like others [9, 41] did not observe the negative influence of barriers on subsequent CRC screening that many have observed [5, 19]. This could be because of differences in how barriers are defined across studies [19] or if they are tailored to a particular test. Some have suggested that barriers may have a test-specific role in CRC uptake [42]; in our study the majority qualified for a stool-based test.

Theory-based CRC screening interventions examining psychosocial predictors of screening in prospective studies are few [9, 14, 41, 43]. Only one of these [41] utilized an SEM approach to examine mediators of CRC screening. In that study, they used the Extended Parallel Process Model to identify mediators of a telephone intervention on uptake of screening colonoscopy among first degree relatives of CRC cases. They observed that the intervention was partially mediated through perceived threat (susceptibility, severity and risk) efficacy beliefs (response efficacy, self-efficacy and barriers), emotions (worry, psychological distress and fear), and behavioral intentions. Direct comparisons with our study are difficult because of differences in the constructs, the population (that population was predominantly non-Hispanic white), risk level (that population was at higher risk) and test (colonoscopy). However, in common with our study, they also observed the important role of self-efficacy and of constructs similar to benefits. The only other studies using an SEM approach have been cross sectional studies that examined correlates of past behavior [40, 44]. As discussed earlier, cross sectional correlates of screening appear to be different to mediators identified in prospective studies [12, 40]. Theory based interventions among populations with significant proportions of Hispanics are few; in one such study predictors were not examined [45], and the other intervention was ineffective [10] but predictors one year afterwards were determined to be self-efficacy and discussion with a provider [43]. They, like us also observed that including all psychosocial constructs in the model improved prediction of screening [43].

Although CRC knowledge had a direct negative influence on screening rates, it also had an indirect positive effect on screening rates through improving the combined overall psychosocial score. In the literature, awareness of the need for CRC screening is reported to be necessary but insufficient for CRC test completion: some studies have found a positive association [9, 46, 47], whereas others have not [43, 48]. Based on our findings and those of others it is apparent that relationship of knowledge to behavior is complex; improved knowledge does not necessarily result in greater uptake of screening. This makes sense when one considers that knowledge acquisition may reflect both positive aspects (e.g. health benefits) as well as negative aspects (e.g. cost or complications) of the behavior. Furthermore, knowledge acquisition may have a complex pattern of influence on different psychosocial variables and the net effect on the behavior may therefore be difficult to predict. Some have suggested that the role of knowledge in screening test completion may not be as important as other psychosocial variables [48] or that its influence may be greater if baseline knowledge is low [9].

All the psychosocial scores including knowledge were improved after the educational intervention except for susceptibility. The psychosocial score was in turn significantly associated with the intervention, knowledge, doctor recommendation for screening and negatively associated with health status. The HBM proposes that a particular health behavior is predicted by six constructs; perceived susceptibility, perceived severity of the condition, perceived benefits, perceived barriers to the behavior, cues to action and self-efficacy to perform the behavior. The ordering or relationship between the variables is not defined [49]. The ACCION intervention targeted all six constructs. Our results indicate that interrelationships between the constructs are important, as combined, they may explain a greater portion of the intervention effect than when considered individually.

Study strengths are that this is one of the first studies to examine multiple psychosocial constructs as mediators of CRC screening in a vulnerable Hispanic population. In addition to testing the hypotheses listed above for the first time, this study also assessed the most effective psychosocial mediators of screening, the specific part of the model which explained maximum variability, multivariate predictors for screening outcome, individual psychosocial scores, and a combined construct, and important psychosocial scores for estimating the psychosocial construct. With these analyses, the total, direct, and indirect effects of the intervention and correlations among post intervention psychosocial responses were also estimated. The findings were further validated by separate SEM analyses including confounders between intervention groups as well. These analyses confirm the robustness of the results obtained in the study. Having done so we were able to identify that psychosocial factors are important mediators for CRC screening uptake and interventions like ACCION, can successfully target constructs to effect screening and the tested conceptual model may be adapted for other cancer screening interventions.

Despite the strengths of this study, there are a number of limitations that should be mentioned. First, the participants in this study were recruited from El Paso and Cameron Counties on the U.S.-Mexico border region of Texas. Therefore, our findings may not be generalizable to other populations (although many of our findings were consistent with previous studies in other populations). Another notable limitation is that the population were uninsured and therefore findings may not apply to those with insurance. Furthermore, only 25 participants were eligible for colonoscopy screening, so these findings may not be applicable to colonoscopy screening. Since there were so few in the colonoscopy group, we were unable to run separate analyses by test type. Another limitation is that there may be unaccounted for mediators that we did not consider, such as defensive processes which have been found to be associated with predictors CRC screening behaviors [50] and may in turn influence CRC screening.

## Conclusion

In summary, we found that the latent psychosocial health construct derived using the post education extended HBM had marginally better predictive ability for screening completion compared to individual post intervention psychosocial measures. Interventions among Hispanic and underinsured populations should consider targeting self-efficacy, perceived benefits, and fatalism in order to improve the uptake of CRC screening. Our study suggests that interventions that change personal beliefs about an individual’s own ability to reduce unhealthy colorectal cancer behaviors, that utilize positive reinforcement, or highlight benefits of adopting healthy behaviors to minimize cancer risk, and that change beliefs to enhance personal power or control to minimize barriers are critical for increasing screening uptake. The challenge is how to facilitate positive changes in these constructs. In our experience key successful approaches in this Hispanic community were developing an understanding of baseline knowledge, beliefs and cultural influences on CRC screening, integrating culturally tailored approaches (language concordance, input from individuals from the community in material development and messaging), and utilizing modelling, vicarious reinforcement in conveying information in a preferred format (in-person, and novella style video). It is essential in any community to do the exploratory work specific to the behavior that is targeted. Future studies should be conducted in more diverse settings, in other Hispanic groups and should focus on individuals receiving colonoscopy screening as well.

## Data Availability

The datasets analyzed during the current study are available from the corresponding author on reasonable request.
